# Complex Transfemoral Access During Transcatheter Aortic Valve Replacement: A Narrative Review of Management, Complexity Scores, and Alternative Access

**DOI:** 10.3390/life15050810

**Published:** 2025-05-19

**Authors:** Ioannis Skalidis, Neila Sayah, Thierry Unterseeh, Thomas Hovasse, Francesca Sanguineti, Philippe Garot, Youcef Lounes, Antoinette Neylon, Mariama Akodad

**Affiliations:** 1 Institut Cardiovasculaire Paris-Sud, Hôpital Jacques Cartier, Ramsay-Santé, 91300 Massy, France; 2 School of Medicine, University of Crete, 71500 Heraklion, Greece

**Keywords:** aortic stenosis, alternative vascular access, complex femoral access, peripheral artery disease, transcatheter aortic valve replacement

## Abstract

Transcatheter aortic valve replacement (TAVR) has become a well-established treatment for severe aortic stenosis across all levels of surgical risk. While transfemoral access remains the default approach, complications arising from vascular access—especially in patients with peripheral artery disease (PAD)—pose significant challenges. Hostile vascular access, characterized by narrow vessel diameters, severe calcification, and tortuosity, complicates the procedure and necessitates alternative strategies. Recent advancements, such as intravascular lithotripsy (IVL), have shown promise in managing severely calcified arteries, improving the feasibility of transfemoral TAVR in patients previously considered ineligible. IVL uses pulsatile sonic waves to fragment arterial calcifications, enhancing vessel compliance and facilitating safe device delivery. Studies have demonstrated that IVL-assisted TAVR improves procedural success and reduces complications in patients with PAD. Additionally, orbital atherectomy, an adjunctive therapy targeting both concentric and eccentric calcifications, may complement the management of complex arterial calcification. The Hostile and passage–puncture scores offer valuable risk stratification tools for predicting vascular complications, aiding in better access site selection. Post-procedural echocardiography, particularly femoral artery sonography, may also play a role in detecting vascular complications early, enabling timely intervention. Finally, alternative access sites are increasingly being explored, with emerging data helping to guide the final access site decision. As TAVR continues to expand into lower risk populations, optimizing vascular access strategies remains essential to improving procedural outcomes. This review highlights the importance of preoperative imaging, endovascular techniques, and post-procedural monitoring in overcoming vascular challenges and ensuring successful TAVR outcomes.

## 1. Introduction

Transcatheter aortic valve replacement (TAVR) has revolutionized the treatment of aortic stenosis, transforming from a complex, high-risk intervention into a widely well-established and standardized procedure [1]. Over the past 15 years, the scope of TAVR has expanded significantly, with indications now extending beyond inoperable and high-risk patients to include intermediate- and low-risk individuals [2].

TAVR has increasingly supplanted surgical aortic valve replacement (SAVR) as the gold standard for elderly patients with severe aortic valve stenosis [3]. However, alongside its clinical success, new challenges have arisen, particularly regarding complications that require prompt recognition and effective management. Vascular access is the critical first step in any TAVR procedure, and various access routes are available, including transfemoral, transaxillary/subclavian, transcarotid, transaortic, transapical, and transcaval [4].

While the first TAVR was performed via an antegrade transseptal approach, the transfemoral retrograde access has become the dominant method, accounting for over 90% of cases in most centers today [5]. Although alternative access strategies, such as transapical, transaxillary, transcarotid, and transcaval approaches, are available, they are generally reserved for cases where transfemoral access is not feasible, due to the inherent procedural complexities and a higher risk of complications [4].

Recent guidelines from the European Society of Cardiology (ESC) recommend TAVR as the first-line treatment for patients over 75 years of age with severe symptomatic aortic valve stenosis, provided transfemoral access is feasible [6]. Similarly, the 2020 American guidelines for valvular heart disease advocate for shared decision-making between SAVR and TAVR for patients aged 65–80 years with symptomatic severe aortic valve stenosis, again contingent on the feasibility of transfemoral access. In cases where transfemoral access is not feasible, the Heart team must consider alternative approaches, including surgical aortic valve replacement or TAVR via transaxillary, transcarotid, transthoracic (transapical or direct aortic), or transcaval access [7]. Currently, 5 to 6% of patients are ineligible for transfemoral TAVR (TF-TAVR) due to peripheral arterial disease (PAD), further highlighting the need for an optimal knowledge and management of alternative access [8].

**Figure 1:**
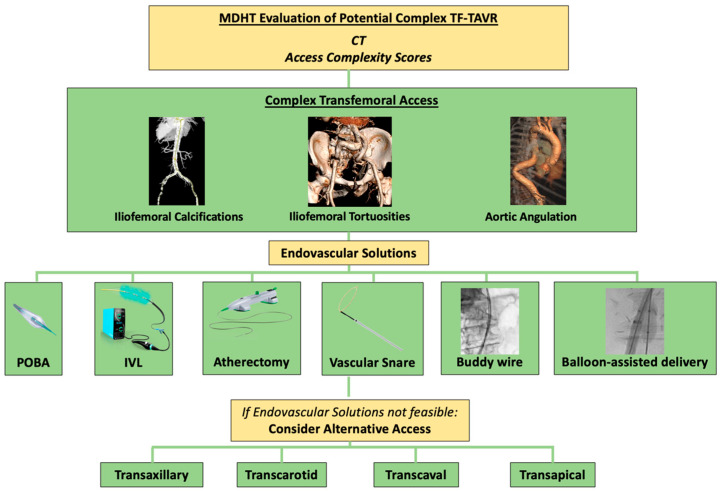
**Proposed algorithm for potential challenges and solutions during complex TF-TAVR.** CT, computed tomography; IVL, intravascular lithotripsy; POBA, plain balloon angioplasty; TF-TAVR, transfemoral access transcatheter aortic valve replacement.

This review will focus on the complexities of transfemoral access for TAVR, including published solutions, management algorithms, and scoring systems, while also providing an overview of alternative access strategies (Figure 1).

## 2. Hostile Vascular Access

Hostile vascular access refers to femoral artery characteristics that make it challenging to use the femoral route for interventions, particularly in procedures like TAVR [9]. The classification of vascular access conditions is based on detailed imaging analysis using computed tomography (CT) scans processed with advanced software. Hostile vascular access is defined by specific anatomical challenges observed during imaging analysis, based on the following criteria:

1. **Narrow Vessel Diameter:**
  o Any segment of the arterial tree with a diameter less than 5.0 mm.
2. **Moderate Narrowing with Severe Calcification or Tortuosity:**
  o Vessel diameter less than 5.5 mm, combined with:
    - Severe calcification (270–360° circumferential calcification),
    - Severe tortuosity (greatest angle of tortuosity <90°).
3. **Combination of Severe Calcification and Severe Tortuosity:**
  o The coexistence of both severe calcification and severe tortuosity along the arterial pathway, regardless of vessel diameter.

These conditions, which are part of the VARC-HBR criteria, may complicate the access and placement of devices, thus categorizing the vascular access as hostile, requiring careful planning and specialized techniques for intervention [10].

## 3. Access Complexity Scores

### 3.1. Hostile Score

The Hostile score is a risk stratification tool used to evaluate iliofemoral anatomy in patients undergoing TAVR, particularly in those with PAD. It incorporates several key factors: the number of segments with significant lesions, total lesion length greater than 100 mm, involvement of atherosclerosis at the aortic bifurcation, presence of significant lesions in tortuous segments, the extent of calcification (greater than 180°), a minimal lumen diameter of less than 5 mm, and any obstruction. A significant lesion is defined as a stenosis greater than 50% (Table 1).

In a study by Palmerini et al., the Hostile score was used to stratify patients with severe PAD undergoing TAVR at 28 international centers [11,12]. The study compared clinical outcomes of TF-TAVR, transthoracic access (TTA), and nonthoracic alternative access (TAA) in these patients. The primary endpoint was the 30-day major adverse event (MAE) rate, which included mortality, stroke/transient ischemic attack (TIA), and major vascular complications. The results showed that both TF-TAVR and TAA were associated with lower 30-day and 1-year rates of MAE compared to TTA. Moreover, lower rates of stroke/TIA were observed with TF-TAVR in patients with low Hostile scores, indicating the score’s potential in predicting access route outcomes.

The SwissTAVI Registry also utilized the Hostile score to predict vascular complications in patients undergoing transfemoral TAVR [13]. In this study, the score was validated for predicting both puncture and non-puncture site complications. Among 2023 patients, the Hostile score was significantly higher in those who experienced vascular complications, particularly non-puncture site complications. Higher Hostile scores, along with factors like female sex and a higher body mass index, were independent predictors of complications. The study showed that the Hostile score had a moderate ability to predict puncture site complications (area under the curve [AUC] 0.554) but a strong predictive value for non-puncture site complications (AUC 0.829). The Hostile score has been shown to be an independent predictor of non-puncture-related vascular complications, such as stroke, following transfemoral TAVR. However, its predictive value for puncture site complications is limited. This discrepancy may be attributed to the score’s emphasis on anatomical features like vessel calcification and tortuosity, which more directly impact non-puncture complications.

### 3.2. Passage–Puncture Score

To address this issue, a novel risk score called the passage–puncture score is developed. This score integrates two main components: the passage feasibility of the iliofemoral arteries (passage score) and the feasibility of the puncture site (puncture score), both based on pre-TAVR CT imaging. The primary goal of this score is to predict vascular complications following TAVR and assist in selecting the optimal femoral access site for the procedure.

The passage–puncture score consists of two key evaluations [14]. The passage score assesses the feasibility of traversing the iliofemoral arteries, while the puncture score evaluates the potential complications at the puncture site itself (Table 1). These assessments are based on preoperative CT imaging and aim to provide a detailed overview of vascular anatomy and potential obstacles. All patients in this study underwent fluoroscopy-guided arterial puncture and closure using a suture-based closure system. The primary endpoint of the study was the rate of vascular complications at discharge, classified as either minor or major according to the Valve Academic Research Consortium-3 definitions [15].

Data from the study of Chen et al., which included 99 patients between September 2020 and June 2021, revealed that the passage score was significantly higher in patients treated by left femoral access compared to those treated by right femoral access (3 vs. 1, *p* < 0.001) [14]. Additionally, the puncture score was significantly different between patients undergoing mid-femoral puncture compared to non-mid-femoral puncture (0 vs. 3, *p* < 0.001). Minor vascular complications occurred in six patients (6%), highlighting the potential benefit of using the passage–puncture score in reducing the risk of these complications.

## 4. Severely Calcified Peripheral Artery Disease Preparation

TF-TAVR is contraindicated in cases of significant PAD where the iliofemoral anatomy cannot accommodate a successful procedure due to the absence of a suitable delivery device [16]. In such cases, alternative strategies must be carefully considered to optimize outcomes.

A detailed preoperative evaluation using CT imaging of the iliofemoral vessels is essential to assess the feasibility of TF-TAVR and to formulate an appropriate treatment plan (Table 2).

### 4.1. Intravascular Lithotripsy

Intravascular lithotripsy (IVL) has recently emerged as a promising therapeutic approach for patients with PAD who are otherwise ineligible for TF-TAVR [17]. The IVL system utilizes pulsatile, low-frequency sonic pressure waves to target and fragment both superficial and deep arterial calcifications. This process does not harm the surrounding non-calcified endothelium or affect the elasticity of the arterial wall, thereby maintaining vascular integrity. The fragmentation of calcifications enhances vessel compliance, enabling the safe advancement of large-bore delivery sheaths necessary for TAVR and reducing the risk of complications such as vessel dissection or rupture.

Several studies have demonstrated the positive impact of IVL-assisted TF-TAVR, particularly in patients with severe iliofemoral vascular disease, showing that this technique can improve procedural success and expand the number of patients eligible for TF-TAVR. Moreover, possible optimal calcium distribution for IVL usage has been documented [18] (Table 3 and Table 4).

A recent study involving 13 patients with severe calcified PAD highlighted the feasibility of IVL-assisted TF-TAVR [19]. In this cohort, IVL was performed after plain old balloon angioplasty (POBA) failure, and it successfully enabled valve delivery in 92.3% of cases. Despite some challenges, such as a single patient experiencing procedural bleeding, no major adverse events were reported during the long-term follow-up. This study suggested that IVL can be a valuable strategy in overcoming the barriers posed by calcified PAD when standard POBA fails, offering a safe and effective means to achieve successful valve deployment in TF-TAVR procedures. However, the authors also noted the need for standardization in defining the severity of PAD and refining indications for upfront IVL use in such cases.

A multicenter study conducted across six European centers (2018–2020) included 108 patients with severe calcific PAD undergoing IVL-assisted TF-TAVR [16]. The target lesions were predominantly located in the common and external iliac arteries (93.5%), with an average minimal lumen diameter of 4.6 ± 0.9 mm and a calcium arc of 318°. IVL-assisted TF TAVR achieved 100% valve delivery success, with a final procedural success rate of 98.2%. Among the complications, there was one perforation and three major dissections that required stent implantation (two covered stents, one bare-metal stent). Access site complications included three major bleedings, and there were three in-hospital deaths (2.8%)—one due to failed surgical conversion after annular rupture, one from cardiac arrest after initial valvuloplasty, and one from late hyperkalemia in renal dysfunction.

Another study compared IVL-assisted TF-TAVR to alternative access approaches, such as transaxillary (TAX) TAVR, in patients with severe iliofemoral calcifications. IVL was employed in 30 patients who presented with severe calcifications precluding standard TF access [20]. This approach demonstrated a higher technical success rate of 93.3% compared to 81.8% for TAX-TAVR. Additionally, the IVL-TAVR group had a significantly lower incidence of complications, including access-related bleeding and vascular complications. These findings suggest that IVL can provide a safer and more effective solution for patients with severe calcified PAD, potentially expanding the pool of candidates suitable for TF access and avoiding the need for more invasive alternative approaches.

In a broader, population-based study analyzing over 129,655 patients undergoing TAVR in the United States, IVL-assisted procedures were increasingly adopted to facilitate TF access in patients with calcified PAD [21]. The study found that IVL TAVR was associated with a higher burden of comorbidities and more complications, particularly vascular issues, compared to non-IVL TAVR. However, despite the increased complication rate, in-hospital death and stroke rates were similar between the two groups. Recent comparative studies have evaluated the efficacy of IVL versus atherectomy. For instance, the ROTA.SHOC trial demonstrated that IVL achieved similar stent expansion compared to rotational atherectomy, with no significant differences in procedural time or contrast volume [22]. Finally, a study by Imran et al. reported that IVL-assisted TAVR patients had a higher burden of comorbidities and experienced more complications, including vascular complications and major bleeding, compared to non-IVL TAVR patients [21]. These findings suggest that while IVL is a promising technique, its benefits may be influenced by factors such as patient selection and operator experience that will affect outcomes.

The ongoing debate regarding the best endovascular treatment for PAD in the context of TF TAVR aligns with these findings. The question of whether IVL offers superior clinical outcomes over traditional POBA needs to be resolved, and the anatomical limitations of IVL, particularly in extreme cases of circumferential calcification, require further exploration. It is also important to consider the role of IVL within a broader treatment strategy that includes other advanced techniques, such as orbital atherectomy, to optimize outcomes and avoid the need for alternative access routes. This brings us to the key unanswered question: which endovascular treatment for PAD is optimal when performing TF TAVR in patients with calcific PAD? Several techniques could be considered, including POBA, calcium modification using IVL, orbital atherectomy, or the “pave and crack” technique for severe circumferential calcifications. While data from the Disrupt PAD III trial comparing POBA and IVL are informative, there remains a lack of direct comparative studies in the TAVR setting. Long-term outcomes of IVL-assisted TAVR are still being evaluated. While short-term results are promising, comprehensive data on durability and long-term efficacy remain limited. However, more research is needed to define the specific indications for IVL use, to compare its outcomes with other calcium modification techniques, and to establish clear criteria for its application in TF TAVR.

### 4.2. Orbital Atherectomy

Orbital atherectomy (OA) has emerged as a valuable adjunctive therapy for TF-TAVR in patients with severely calcified iliofemoral arteries [23]. This is particularly crucial in cases where peripheral arterial disease and extensive calcification hinder safe access for valve delivery. Unlike other modalities such as IVL, which primarily targets concentric calcifications, orbital atherectomy effectively debulks both concentric and eccentric calcifications. The Stealth 360^®^ peripheral OA system uses a diamond-coated crown that delivers 360° centrifugal force, allowing for differential sanding of calcium and minimizing the risk of vessel injury. This method is particularly advantageous in cases where calcium buildup is uneven, and it significantly enhances the feasibility of safe vascular access, making it possible to perform TF-TAVR in previously high-risk patients.

Orbital atherectomy is effective in intimal or intraluminal deposits, but it does not significantly modify medial calcifications [24]. The target vessel diameter ranges from 5 mm to 10 mm, utilizing a 0.014″–0.018″ ViperWire ™ and maintaining compatibility with a 6F system. A final contrast angiography should be performed to rule out significant vascular injuries at the iliac and/or femoral arteries. A key limitation of both IVL- and orbital-atherectomy-assisted TF-TAVR is that neither technique can be applied to the puncture site itself. When the access site is heavily calcified or diseased, the risk of vascular closure device failure remains significantly elevated.

## 5. Severe Tortuosity Management

### 5.1. Aortic Angulation

In cases of severe aortic angulation, the bilateral buddy wire technique can be invaluable for facilitating the advancement of the transcatheter heart valve (THV). When encountering difficulty with a stiff wire due to tortuosity or a kink, introducing a second stiff wire into a contralateral catheter can help straighten the vessel and enable smooth THV navigation [25].

The buddy wire in the catheter plays a key role in straightening the vessel, ensuring the smoother passage of the THV. Softer and more steerable delivery systems may also assist in advancing the device more easily. However, attempting to force the system through extreme angulation carries a risk of aortic rupture, which can be mitigated by preparing for potential endovascular stent grafting if necessary.

The snare technique can also be an effective strategy to facilitate TAVR in patients with highly angulated aortas [26]. A snare catheter is typically introduced via the contralateral femoral artery or radial artery and advanced through a 7-F sheath. The snare captures the prosthesis within the aortic arch or at the aortoiliac bifurcation, securing it at the middle third of the device to generate the necessary tension for smooth passage through the aortic valve. Once proper positioning is achieved, the snare is carefully withdrawn, allowing for controlled prosthesis deployment. Key technical considerations include selecting an appropriate vascular access route, ensuring precise snare engagement to maintain stability, and applying controlled traction to facilitate advancement without excessive force.

### 5.2. Iliofemoral Tortuosity

Iliofemoral tortuosity presents significant challenges during TAVR, often hindering the advancement of sheaths and prostheses. The buddy wire technique, utilizing two parallel stiff guidewires (e.g., Lunderquist^®^ extra-stiff wire guide), effectively straightens the vascular anatomy to facilitate device deliverability while minimizing arterial trauma [27]. This approach involves advancing a second stiff guidewire alongside the first through a guiding catheter, then allowing for controlled sheath progression on one of the stiff wires while maintaining the second wire external to the sheath. By reducing arterial wall contact with the TAVR delivery system and avoiding additional arterial punctures, this technique enhances procedural safety and efficiency. However, potential risks include wall dissections in heavily calcified and tortuous iliac arteries, the widening of the access site in fragile femoral arteries, and a theoretical stroke risk if the wire is positioned within the aortic arch. Proper wire positioning distal to the left carotid artery mitigates this latter concern (Videos S1–S3).

Another technique to facilitate sheath advancement in challenging TF-TAVR cases is the balloon-assisted sheath insertion. This method involves inflating a balloon to modify the contact point between the sheath tip and the graft wall, allowing for smoother passage through tortuous or stenotic segments [28]. Unlike the balloon anchoring method, it avoids the risk of sheath expansion, and unlike the pull-through technique, it does not require additional vascular access. Given its simplicity and safety, this technique may be a useful alternative for difficult transfemoral interventions.

## 6. Management of Aortic Wall Pathology (Aneurysm–Thrombus)

Aortic aneurysms and thrombi have been investigated in studies assessing their impact on TAVR. A retrospective analysis found that 9.5% of TAVR patients had concomitant aortic aneurysms, with no significant difference in vascular complications or MACE at six months compared to those without aneurysms [29]. However, another study reported a 1.97-fold increase in in-hospital mortality and a higher incidence of intraoperative cardiac arrest in patients with aortic aneurysms [30]. A prospective study investigating aortic wall thrombus (AWT) in patients undergoing TAVR found that severe AWT, based on a specific score, identified on pre-procedural multidetector computed tomography, was significantly associated with an increased risk of thromboembolic events. Specifically, patients with severe AWT had an 8.48-fold higher likelihood of experiencing procedural thromboembolic complications, including ischemic stroke, blue toe syndrome, bowel ischemia, or other solid organ infarctions. The presence of severe AWT was also linked to a fivefold increased risk of stroke (Odds Ratio: 5.66) and a fourfold higher risk of procedural death (Odds Ratio: 4.66) [31].

## 7. Complex Puncture Site Management

Complex femoral puncture sites in TAVR procedures pose significant challenges due to variations in vascular anatomy and patient-specific factors. High common femoral artery bifurcation (HCFAB) is present in approximately 30% of TAVR patients and can complicate the puncture, increasing the risk of suprainguinal puncture and retroperitoneal hemorrhage during large-bore arteriotomy [32]. This is because the distance between the inguinal ligament (IL) and the bifurcation is often reduced, leaving little margin for error. A study analyzing 150 TAVR patients showed that the average IL-to-bifurcation distance in patients with HCFAB was only 7 mm, highlighting the importance of careful puncture site planning. Ultrasound, combined with palpation of the anterior superior iliac spine and pubic tubercle and fluoroscopy, is recommended to reduce the risk of high-stick punctures in these cases. Furthermore, the superficial femoral artery (SFA) may serve as an alternative access route for TAVR, particularly in patients with severe obesity (BMI > 40), where standard transfemoral access at the common femoral artery may be challenging or unsafe due to excess adipose tissue, vessel depth, or anatomical constraints.

Vascular calcification also plays a critical role in complicating femoral puncture. The CHOICE-CLOSURE trial demonstrated that patients with severe anterior calcification had higher rates of vascular complications, including both major and minor access site complications [33]. Although calcification did not significantly affect the choice of closure strategy, the presence of severe calcification necessitated increased caution when choosing the puncture site and closure technique. The study emphasized the need for tailored access strategies in these patients to minimize complications.

Obesity, though traditionally a concern in other vascular procedures, does not appear to uniformly increase the risk of vascular complications in TAVR. A retrospective analysis of over 1100 TAVR patients found no correlation between obesity and the rate of vascular complications, suggesting that TAVR can be safely performed in patients with a high BMI [34]. However, other studies have reported an increased risk of vascular complications in obese patients undergoing TAVR, highlighting the need for careful patient selection [35]. As always, thorough preoperative imaging, including CTA, should be employed to assess the iliofemoral anatomy and guide puncture site selection.

Finally, echo-guided puncture plays a pivotal role in improving puncture accuracy and reducing complications, particularly in cases with challenging anatomy or significant calcification [36]. The use of real-time echocardiographic guidance provides enhanced visualization and precise needle placement, which is crucial for minimizing vascular injury and ensuring procedural success in complex femoral access sites.

## 8. Alternative Access Sites for TAVR

The 2022 European TAVI Pathway Survey, which analyzed data from 27,223 patients undergoing TAVR across 26 European countries, revealed that percutaneous transfemoral access remains the dominant approach, used in 99% of cases [37]. For alternative access, the transaxillary route is the most commonly preferred, chosen in 50% of cases, with a majority (65%) opting for surgical cutdown rather than a direct percutaneous approach (35%). Transapical access is selected in 6%, 9%, and 12% of cases as the first, second, and third alternative access sites, respectively. The transcarotid approach, when used, overwhelmingly relies on surgical cutdown (94%). Among alternative access routes, transvenous access is the least favored, with transcaval access being the most common (69%). The following section briefly presents the various alternative access sites for TAVR.

### 8.1. Subclavian/Transaxillary Access (TAx)

Transaxillary access is now the most commonly used alternative access site for TAVR, as per the 2019 STS-ACC TVT Registry, with usage increasing from 20% in 2015 to 49% in 2017 for non-transfemoral cases. The axillary and subclavian arteries, with a diameter comparable to the iliofemoral artery, are favorable for access due to their more favorable anatomical characteristics, such as less pronounced atherosclerosis compared to the femoral vessels.

Pre-procedural screening for transaxillary access includes multidimensional computed tomography (MDCT) imaging to evaluate vessel size and characteristics. The minimal luminal diameter must be greater than 5 mm for self-expanding valves and 5.5 mm for balloon-expandable valves, with no severe calcification or excessive tortuosity. Both surgical and percutaneous approaches are used for axillary access as well as IVL use. A meta-analysis of five studies (1903 patients) compared transfemoral and Tax approaches using the CoreValve device. The TAx group had more comorbidities but showed no significant differences in vascular complications, aortic regurgitation, permanent pacemaker implantation, or mortality at 30 days and 1 year [38]. Van Wely et al. recently compared the outcomes between patients undergoing TAVR with TF-TAVR and TAx access using self-expanding devices and compared 354 TAx-TAVR patients with 5980 TF-TAVR patients using propensity score matching [39]. No significant differences were found in 30-day (5% vs. 6%, *p* = 0.90) or 1-year (20% vs. 16%, *p* = 0.17) mortality. Myocardial infarction was more frequent in the TAx group (4% vs. 1%, *p* = 0.05), while permanent pacemaker implantation was lower (12% vs. 21%, *p* = 0.001). Stroke rates were similar (TAx 2% vs. TF 3%, *p* = 0.19).

### 8.2. Transcarotid Access

Introduced in 2010 in France, transcarotid access is typically used in patients with severe iliofemoral and subclavian artery tortuosity. While initial concerns about increased stroke risk limited its use, improved techniques have lowered stroke rates. Preoperative evaluation of the carotid artery is essential, including ultrasonography and MDCT, to assess vessel characteristics and potential risks. A 6.5 mm diameter is the minimum acceptable size for the carotid artery. The access is typically obtained via a 4 cm incision near the sternocleidomastoid muscle, followed by sheath insertion using the Seldinger technique. The carotid artery is then clamped, and the arteriotomy is repaired post-procedure. Several studies have explored the safety and outcomes of the transcarotid (TC) approach as an alternative access site for TAVR. A meta-analysis by Usman et al. summarized the incidence of various outcomes in TC TAVR, noting a decline in stroke and TIA rates over time, likely due to improved operator experience [40]. A recent meta-analysis comparing TC TAVR with other access sites found that TC had higher short-term mortality but lower vascular complications than transfemoral access [41]. Importantly, there was no significant increase in cerebrovascular accidents with TC access compared to other routes. As operator experience with TC TAVR has grown and evidence supporting its safety and efficacy has strengthened, TC has become a preferred alternative access site for some centers [42]. Leclercq et al. evaluated vascular complications after TF-TAVR using an exclusive open surgical access strategy in 396 patients. The procedures used 16Fr to 20Fr sheaths with SAPIEN XT (72.7%) and Medtronic Core Valve (27.3%). Major and minor vascular complications occurred in 2.3% and 4%, with life-threatening or major bleeding in 4.6%. VC led to longer hospital stays (7 vs. 5 days, *p* < 0.001) but did not impact 1-month (6.6%) or 1-year (17.2%) mortality [43].

### 8.3. Transcaval Access

Transcaval access, introduced in 2010, provides a unique approach by creating an arteriovenous fistula between the inferior vena cava (IVC) and the abdominal aorta. The procedure starts with the placement of an introducer sheath in the femoral vein, followed by the advancement of an electrosurgical guidewire into the IVC and then into the aorta. This approach has expanded to include transcatheter aortic repair, temporary mechanical circulatory support, and pediatric interventional cardiology [44]. Post-delivery, the transcaval tract is closed using a nitinol occluder device, and any residual aorto-caval fistulas are typically addressed with minimal intervention. Though transcaval access has shown promise, it remains less commonly used than other alternatives due to the complexity of the procedure.

In studies of clinical outcomes, transcaval access has been shown to be a safer approach compared to other alternative access methods. Barbash et al. examined 185 patients, with 12% undergoing transcaval TAVR and 82% undergoing alternative access TAVR [45]. Their findings revealed a lower rate of acute kidney injury in the transcaval group and shorter hospital stays, though there was no difference in early or 30-day mortality. Lederman et al. performed a meta-analysis comparing transcaval access with transaxillary access and found that stroke rates were lower with transcaval access, while bleeding rates were similar across eight U.S. centers [46]. A recent systematic review comparing transcaval TAVR with supra-aortic access (including both TC and transaxillary approaches) reported no significant differences in in-hospital or 30-day all-cause mortality, major bleeding, blood transfusions, major vascular complications, or kidney injury [47]. Interestingly, transcaval TAVR showed a trend toward a lower rate of neurovascular complications, though it did not reach statistical significance [47].

### 8.4. Rare Access (Transapical Access–Transaortic)

Transapical TAVR, first performed in 2005, was initially favored due to its ability to accommodate large delivery systems regardless of iliofemoral disease. The procedure involves a mini-thoracotomy in the fifth intercostal space, followed by left ventricular apex puncture, sheath introduction, and valve deployment, with suturing to ensure hemostasis [48]. However, high mortality and morbidity rates have led to its decline, with a 30-day mortality of 8.8% and a 1-year mortality of 25.6%. By 2019, transapical access accounted for only 0.3% of cases, largely replaced by safer alternatives [49]. Despite this, it remains useful in select cases, such as double valve-in-valve procedures. Some high-volume centers have reported improved outcomes, with D’Onofrio et al. demonstrating 75% survival at 2 years and 44% at 5 years, while machine learning models have helped refine risk prediction for better patient selection [50].

Transaortic access, performed via partial sternotomy or mini-thoracotomy through the second to fourth intercostal space, provides another alternative. The technique involves purse-string suture placement, needle access, dilation, sheath delivery, and valve deployment under rapid pacing and imaging guidance, typically under general anesthesia. Observational data suggest transaortic TAVR has lower mortality than transapical, but studies indicate higher 30-day complications, particularly major bleeding. A meta-analysis of 34 studies, encompassing over 32,000 patients, found both transaortic and transapical access routes doubled the risk of 30-day mortality compared to transfemoral access [8]. Given these findings, transfemoral or extrathoracic approaches (such as transaxillary, transcarotid, or transcaval) are generally preferred whenever feasible due to their superior safety and lower complication rates.

## 9. Conclusions

The management of transfemoral access during TAVR requires a multimodal approach that incorporates advanced imaging, specialized risk scores and tools, and risk stratification techniques to overcome the complexities associated with vascular access. Innovations like IVL and orbital atherectomy have enhanced the feasibility of transfemoral access, even in patients with severe PAD, reducing the need for more invasive alternatives. The integration of access complexity scores, such as the Hostile score and passage–puncture score, has further refined patient selection and optimized procedural outcomes. However, as the TAVR field continues to evolve, ongoing research is needed to refine these strategies, evaluate the comparative effectiveness of different techniques, and establish clear guidelines for the use of alternative access routes.

## Figures and Tables

**Table 1:** Passage, puncture, and Hostile score variables.

Score	Variable	Description	Points/Format
Hostile Score	Number of segments with significant disease	Each affected segment	1 point per segment
	Presence of obstruction	100% stenosis in any segment	2 points
	Iliac disease involving the aortic bifurcation	Atherosclerotic disease at aortic bifurcation	0.5 points
	Lesion in tortuous segment (tortuosity > 90°)	Significant disease in tortuous segments	1 point per segment
	≥180° arc calcified lesion	Significant calcified lesion with an arch ≥ 180°	1 point per lesion
	Lesion length ≤ 100 mm	Short-segment calcification	1 point
	Lesion length 101–200 mm	Intermediate-length lesion	2 points
	Lesion length > 200 mm	Long lesion segment	3 points
	Minimal lumen diameter 3.1–5.0 mm	Mild narrowing	0.5 points
	Minimal lumen diameter 0.1–3.0 mm	Severe narrowing	1 point
Passage Score	Minimal lumen diameter	Narrowest point of the access artery	0 (favorable), 1 (challenging), 2 (unfavorable)
	Calcification length	Length of calcified segments along the artery	0 (favorable), 1 (challenging), 2 (unfavorable)
	Maximal calcification thickness	Thickness of the most severe calcified plaque	0 (favorable), 1 (challenging), 2 (unfavorable)
	Vessel tortuosity	Presence of double iliac sign or high tortuosity index (with calcification)	0 (favorable), 1 (challenging), 2 (unfavorable)
**Puncture Score**	Bifurcation height	Position of femoral bifurcation relative to puncture site	0 (favorable), 1 (challenging), 2 (unfavorable)
	Calcification orientation	Circumferential location and severity of calcifications	0 (favorable), 1 (challenging), 2 (unfavorable)
	Calcification-free vessel length	Distance from puncture site to nearest calcium	0 (favorable), 1 (challenging), 2 (unfavorable)

**Table 2:** Advantages and disadvantages of different options in calcified peripheral artery disease preparation.

**Technique**	Advantages	Disadvantages
**Plain Old Balloon** **Angioplasty (POBA)**	High immediate technical success	High rates of flow-limiting dissections
	Easy to use	Vascular recoil and residual stenosis
	Efficacious for lesions < 100 mm	High restenosis rate
	Vessel tortuosity	Presence of double iliac sign or high tortuosity index (with calcification) 2
**Atherectomy**	Useful for vessel preparation before balloon or drug-covered balloon angioplasty	Risk of distal embolization
		Less operator familiarity
	Reduces inflation pressures and risk of dissections	High procedural cost
		Not widely available
**Intravascular Lithotripsy (IVL)**	Useful for vessel preparation before balloon or drug-covered balloon angioplasty	Each affected segment
	Reduces inflation pressures and risk of dissections	Requires more technical expertise
		Less operator familiarity

**Table 3:** **Key pre-procedural CT measurements for IVL-assisted TF-TAVR.** CT, computed tomography; IVL, intravascular lithotripsy; MLD, minimal luminal diameter; TAVR, transcatheter aortic valve replacement; TF, transfemoral.

**Parameter**	**Measurement/Condition**	Clinical Relevance
**Minimal Luminal Diameter (MLD)**	Reported at site of most critical stenosis	Determines feasibility of IVL
**Maximal Luminal Diameter**	Measured perpendicular to the vessel axis	Helps calculate mean lumen diameter
**Mean Luminal Diameter**	(Maximal diameter + minimal diameter)/2	Provides overall vessel dimension
**Vessel Area**	Measured at stenotic site	Assesses severity of narrowing
**Vessel Tortuosity**	Evaluated along the iliofemoral course	High tortuosity may increase procedural complexity
**Calcification Arc**	360° or 270° (horseshoe-shaped)	Affects vessel expandability for sheath insertion
**Calcification Length**	Localized (<20 mm) vs. diffuse (>20 mm)	Influences MLD requirements for IVL eligibility
**Calcium Blooming Artifacts**	Can lead to overestimation of calcification severity	Requires windowing correction for accuracy
**Femoral Artery Puncture Site**	Presence of an anterior calcium-free window	Crucial for vascular closure and sheath insertion

**Table 4:** **IVL feasibility based on calcification pattern and severity.** IVL, intravascular lithotripsy; MLD, minimal luminal diameter; TAVR, transcatheter aortic valve replacement; TF, transfemoral.

**Calcification Pattern**	**Minimal Luminal Diameter Requirement**	**Suitability for IVL-Assisted TF-TAVR**
**Localized Calcification (<20 mm length)**		
−360° Circumferential	≥4.0 mm	**Suitable**
−270° Horseshoe-Shaped	≥3.0 mm	**Suitable**
**Diffuse Calcification (>20 mm length)**		
−360° Circumferential	≥4.5 mm	**Suitable**
−270° Horseshoe-Shaped	≥3.5 mm	**Suitable**
**Severe Calcification with MLD < 3.0 mm**	<3.0 mm	**Not suitable for IVL**, consider alternative access
**Irregular or Extensive Eccentric Calcification**	Severe asymmetric calcium distribution	**Not optimal**, higher procedural risk

## Supplementary Materials

The following supporting information can be downloaded at: https://www.mdpi.com/article/10.3390/life15050810/s1, Video S1: Tortuosity of the iliofemoral arteries prevents the advancement of the 0.035″ stiff wire; Video S2: The buddy wire technique, using two Lunderquist wires in parallel, facilitates the advancement of the 14F sheath and the valve; Video S3: Restoration of the original shape of the iliac arteries at the end of the procedure without complications.

## Abbreviations

ESC	European Society of Cardiology
IVL	Intravascular Lithotripsy
MAE	Major Adverse Event
OA	Orbital Atherectomy
PAD	Peripheral Artery Disease
POBA	Plain Old Balloon Angioplasty
SAVR	Surgical Aortic Valve Replacement
TAVR	Transcatheter Aortic Valve Replacement
TAX	Transaxillary
TF-TAVR	Transfemoral Transcatheter Aortic Valve Replacement
